# Estimation of Genetic Effects and Genotype-Phenotype Maps

**DOI:** 10.4137/ebo.s756

**Published:** 2008-06-28

**Authors:** Arnaud Le Rouzic, José M. Álvarez-Castro

**Affiliations:** 1 Center for Ecological and Evolutionary Synthesis, University of Oslo, Oslo, Norway; 2 Department of Animal Breeding and Genetics, Swedish University of Agricultural Sciences, Uppsala, Sweden

## Abstract

Determining the genetic architecture of complex traits is a necessary step to understand phenotypic changes in natural, experimental and domestic populations. However, this is still a major challenge for modern genetics, since the estimation of genetic effects tends to be complicated by genetic interactions, which lead to changes in the effect of allelic substitutions depending on the genetic background. Recent progress in statistical tools aiming to describe and quantify genetic effects meaningfully improves the efficiency and the availability of genotype-to-phenotype mapping methods. In this contribution, we facilitate the practical use of the recently published ‘NOIA’ quantitative framework by providing an implementation of linear and multilinear regressions, change of reference operation and genotype-to-phenotype mapping in a package (‘noia’) for the software R, and we discuss theoretical and practical benefits evolutionary and quantitative geneticists may find in using proper modeling strategies to quantify the effects of genes.

## Introduction

Quantitative genetics aim at providing models that describe the genetic architecture of complex phenotypic traits. These so-called multifactorial characters are generally underlain by several genes and by environmental factors, that can potentially interact in many ways. One of the most insightful applications of dissecting the genetic architecture of a quantitative trait, e.g. through QTL mapping experiments, is to determine individual genetic effects. However, the proper quantification of allelic effects from experimental data, as well as the description of the Genotype-to-Phenotype (GP) map, remains challenging. In particular, it is well known that the effect of a particular allele often depends on the genetic background, i.e. the genotype at other loci.

Genetic interactions (or epistasis), when measured from a polymorphic population, change with allelic frequencies. Fisher (1918) first developed a mathematical framework describing how genetic effects of allele substitutions in populations affected the mean phenotype. Kempthorne (1954) and Cockerham (1954) extended Fisher’s work to account for epistasis. This ‘statistical epistasis’ (*sensu* Cheverud and Routman, 1995) provides information about e.g. the evolutionary properties of a particular population, but is of little interest for the geneticist who is more focused on the effect of genetic interactions in a given genotypic background —‘physiological’ (Cheverud and Routman, 1995) or ‘functional’ (Hansen and Wagner, 2001) epistasis. The mathematical and statistical issues underlying a proper quantification of genetic effects are not trivial, and keep on generating a respectable amount of literature nowadays (Kao and Zeng, 2002; Yang, 2004; Zeng et al. 2005; Wang and Zeng, 2006).

In a recent contribution, Álvarez-Castro and Carlborg (2007) described a general framework, the Natural and Orthogonal InterActions (NOIA) model, which provides mathematical tools to compute and manipulate genetic effects and GP maps. In particular, NOIA ensures that the statistical estimation of genetic effects remains orthogonal regardless of the genotypic frequencies at each locus in the population under study, i.e. even if the population deviates from expected Hardy-Weinberg proportions. This property makes the model useful in a broad range of situations, including the study of natural populations, and should be prefered to more constrained models (such as those forcing the frequencies to fit to an F_2_ population or to Hardy-Weinberg proportions). Furthermore, once the estimates of genetic effects have been obtained using the proper statistical formulation of genetic effects, researchers might want to express them in a different way, e.g. describing the average effects of allele substitutions at a different population than the sample under study, or describing natural effects of allele substitutions from an individual genotype—i.e. functional estimates of genetic effects as described above. NOIA provides tools to transform the estimates obtained from the data into these other estimates with different useful meanings (Álvarez-Castro and Carlborg, 2007; Álvarez-Castro et al. 2008).

Geneticists may benefit from such theoretical improvement in a broad range of situations, including (i) when estimating the genetic architecture of a trait after a QTL mapping experiment, and (ii) when genotyping loci known to underlie a complex trait in a natural population. The aim of this communication is to present statistical and bioinformatic tools designed to use NOIA in practice. First, we propose to extend the NOIA framework to handle the decomposition of the genetic variance and the computation of confidence intervals of genetic effects. We then describe how to simplify the analysis of complex GP maps through the multilinear model of genetic interactions proposed by Hansen and Wagner (2001). Finally, we present an implementation of the NOIA model to obtain genetic effects at any location in the genome in a user-friendly package for the software R ( R Development Core Team, 2007), and we illustrate the benefits provided by this framework.

## Model

### The NOIA model

#### General framework

Zeng et al. (2005) proposed to link the Genotype-to-Phenotype (GP) map (i.e. the vector genotypic values **G**) to the vector of genetic effects **E** by:

(1) $$G=S_{R}\cdot E_{R}$$

S*_R_* being the genetic-effect design matrix for the reference point *R*. If the model is orthogonal, genetic effects are ‘statistical’, and the reference point is *μ*, the mean of the population. According to Álvarez-Castro and Carlborg (2007), in the case of a single-locus model with two alleles *A* and *a* (and three genotypes, noted *AA* = 1, *Aa* = 2 and *aa* = 3, which respective frequencies are *p*_1_, *p*_2_ and *p*_3_), the orthogonal decomposition of genetic effects is:

(2) $$G=\left(\begin{array}{c} G_{1}\\ G_{2}\\ G_{3} \end{array}\right)=\left(\begin{array}{ccc} 1 & -p_{2}-2p_{3} & -\frac{2p_{2}p_{3}}{p_{1}+p_{3}-{\left(p_{1}-p_{3}\right)}^{2}}\\ 1 & 1-p_{2}-2p_{3} & \frac{4p_{1}p_{2}}{p_{1}+p_{3}-{\left(p_{1}-p_{3}\right)}^{2}}\\ 1 & 2-p_{2}-2p_{3} & -\frac{2p_{1}p_{2}}{p_{1}+p_{3}-{\left(p_{1}-p_{3}\right)}^{2}} \end{array}\right)\times \left(\begin{array}{c} \mu \\ \alpha \\ \delta \end{array}\right)$$

The genetic effects α and δ correspond to the additive and dominance effects. Assuming linkage equilibrium, the extension of equation 2 to any number of loci through simple matrix algebra is straightforward (Álvarez-Castro and Carlborg, 2007).

When a specific genotype is chosen as a reference, the corresponding effects will be ‘functional’, and they no longer depend on genotypic frequencies in the population. Genetic effects from a specific genetic background reference (*R_B_*), E_*R_B_*_, can be obtained by the ‘change of reference’ operation:

(3) $$E_{R_{B}}=S_{R_{B}}^{-1}\cdot S_{R}\cdot E_{R}$$

where S_*R_B_*_ is the genetic-effects desing matrix fitting the new reference point *R_B_* (see Álvarez-Castro and Carlborg, 2007 for more details).

#### Linear regression

The survey of a natural or an artificial population will bring both phenotypic and genetic information. When the location of the genetic factors involved in the trait under study is known, each individual *i* of the population will be identified by its phenotype *y_i_* and its genotype *g_i_*. According to most models in quantitative genetics, the phenotype results from a combination of genetic and environmental factors, such as, *y_i_* = *G _g_i__* + *e_i_* where *e_i_,* a factor due to environmental noise, is a random, normally-distributed variable. Therefore, provided a sufficient population size, the GP map can be evaluated by a linear regression:

(4) Y=Z·G+e

where **Z** is a matrix that reflects the genotype of each observed individual, whose phenotypes are **Y** (Álvarez-Castro and Carlborg, 2007; Álvarez-Castro et al. 2008). From equation 1, the statistical genetic effects can be evaluated by a linear regression as well:

(5) $$Y=Z\cdot S_{S}\cdot E_{S}+e$$

#### Variance computation

The variance explained by a specific genetic effect depends on the frequency of the different genotypes in which this effect is involved. If **V** is the vector of genetic variances, and **F** the vector of genotypic frequencies, then

(6) $$V=F\cdot (S\circ S)\cdot {\left(E\circ E\right)}^{T}$$

where *^T^* denotes the transposition operation, and ○ is the Hadamard product (i.e. pairwise product of the elements of the two matrices).

The decomposition of variance components, a classical procedure in quantitative genetics, can be performed in a straightforward way by summing up the variances of the effects at the same level: the additive variance will be the sum of the variances of single additive effects, etc. If the model is orthogonal, then the sum of all effect variances should add up to the total genetic (explained) variance.

Another useful result is the computation of confidence intervals for the estimates of genetic effects and genotypic values. The standard deviation of the (linear or non-linear) regression coefficients, σ*_E_*, are provided directly by R (R Development Core Team, 2007). The equivalent standard errors for the GP map can be derived from Equation 1:

(7) $$\sigma_{G}^{2}=(S\circ S)\cdot \sigma_{E}^{2}.$$

### The multilinear model in the NOIA framework

#### The multilinear model of genetic interactions

The multilinear model (Hansen and Wagner, 2001; Carter et al. 2005) provides a way to describe complex multilocus GP maps through a reduced number of parameters, given some approximations. The underlying hypothesis is that epistatic interactions result from a scaling by a parameter ɛ. Considering two independent allelic substitutions at two different loci, and *i* and *j* their respective effects on the phenotype, the multilinear model predicts that both substitutions occuring together will result in a genotypic value of: *i* + *j* + ɛ*_ij_* · *i* · *j*.

#### Multilinear regression

The general model provided by NOIA generates 3*^L^* genetic effects for *L* loci, which becomes increasingly difficult to handle with high number of loci. The multilinear approximation leads to a simplification of the models by reducing the number of parameters. For instance, for a 2-locus (*A* and *B*) model,

(8) $$E=\left(\begin{array}{c} \mu \\ \alpha_{A}\\ \delta_{A}\\ \alpha_{B}\\ \alpha \alpha_{AB}\\ \delta \alpha_{AB}\\ \delta_{B}\\ \alpha \delta_{AB}\\ \delta \delta_{AB} \end{array}\right)=\left(\begin{array}{c} \mu \\ \alpha_{A}\\ \delta_{A}\\ \alpha_{B}\\ \alpha_{A}\cdot \alpha_{B}\cdot {ɛ}_{AB}\\ \delta_{A}\cdot \alpha_{B}\cdot {ɛ}_{AB}\\ \delta_{B}\\ \alpha_{A}\cdot \delta_{B}\cdot {ɛ}_{AB}\\ \delta_{A}\cdot \delta_{B}\cdot {ɛ}_{AB} \end{array}\right)$$

the right-hand side vector contains only 6 parameters to estimate, compared to the 9 of the full model. Transferring this in equation 5 leads to a non-linear regression. Its implementation in the software R (R Development Core Team, 2007), through the *nls* procedure, showed that the numerical convergence was generally not problematic when proper starting values are provided (calculated from the result of a linear regression).

### Implementation

The NOIA framework has been implemented as a fully documented package for the free software R, which is available on most common operating systems including Linux, Microsoft Windows and Macintosh. The linear and non-linear regressions rely repectively on the *lm* and *nls* (library *stats*) functions of R. The noia package is released under the General Public Licence, and can be freely downloaded from the CRAN depository http://cran.r-project.org/web/packages/noia/index.html.

## Results and Discussion

### From the data to the Genotype-Phenotype map

#### Dataset

In the ideal situation, the exact genotype is known at the expected location of the genetic factors that are thought to influence the trait. This reduces uncertainties and increases the power of the analysis. However, in many cases, the genetic information consist of markers that are close to, but not exactly at the quantitative trait locus. In this latter situation, it is possible to calculate the probability of the genotype at the locus from the genotype at flanking markers, knowing the recombination rate between markers, the most common method being the Haley-Knott regression (Haley and Knott, 1992). The extension of the NOIA framework to such a situation is explained in detail in Álvarez-Castroet et al. (2008).

**Figure 1:**
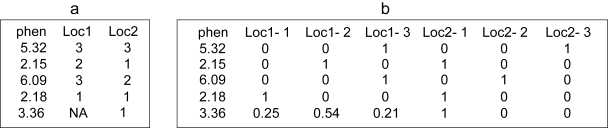
Illustration of data formatting. Part a provides an example of a data set in which the genotypes of individals are fully known (or, alternatively, totally unknown and considered as missing data); 1 and 3 stand for the homozygotes (e.g. ‘AA’ and ‘aa’) and 2 for the hererozygote. Part b illustrates a second kind of data set in which the genotypes are defined by their probabilites. In this example, part b is the exact equivalent of part a (and then, the frequency of the ‘known’ genotypes is always 1), but in practice, especially when the data result from a Haley-Knott regression, the probabilities, computed from the genotypes at flanking markers, may be intermediate. Missing values (‘NA’) are allowed in type a data sets, and are replaced by genotypic probabilities equal to genotypic frequencies in the rest of the population (here, close to 0.25, 0.5, and 0.25 since the population is an F_2_). The Z matrix used for the regression (equation 5) is computed from a ‘type b’ data set, meaning that if ‘type a’ data is provided, it is turned into ‘type b’ before the genetic regression.

The noia package provides two kinds of possible input data sets that correspond to these two situations (Fig. 1). The exact genotypes are provided as an array of *L* columns, *L* being the number of loci (Fig. 1a). The genotypes are coded such as 1and 3 are homozygotes, while 2 stands for the heterozygous genotype. Missing data are allowed, each missing genotype being weighted according to its frequency in the rest of the population. The partial genotypic information are provided by a 3*L* columns array, in which each of the *L* loci is represented by a set of 3 columns standing for the probabilities of the three genotypes 1, 2 and 3, the sum of these being 1 (Fig. 1b).

#### Genetic effects and GP map

The linear and multilinear regression functions (linearRegression and multilinearRegression respectively) estimate genetic effects in the NOIA statistical framework, and display the estimated values of genetic effects, the corresponding part of genetic variance explained, the standard deviation of the estimate and the probability that the corresponding effect is 0 (as provided by the *lm* and *nls* functions in R). Table 1 presents an example from a simulated population, with both statistical and functional genetic effects (the full script is provided in the Appendix).

**Figure 2:**
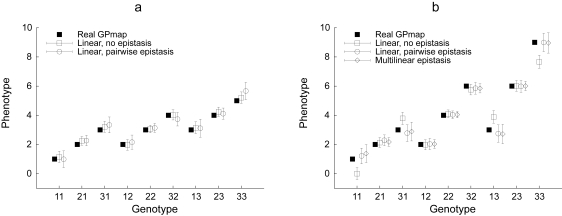
Accuracy of GP map predictions. The estimate of genotypic values, as well as their 95% confidence intervals, are shown for two different tow-locus Genotype-Phenotype maps (a: no epistasis, b: multilinear epistasis). Results are derived from simulated F_2_ populations of size *N* = 200 (the script is provided in the Appendix). Predictions are satisfactory, except if the model cannot handle the complexity of the map (marginal effect model on an epistatic map). Confidence intervals are smaller when the genotypic value is estimated from a frequent genotype in the population (the most frequent genotype in an F_2_ being 22), and when the model has less degrees of freedom (such as in one-locus models). 95% confidence intervals are estimated from the standard error (SE) by *CI* = 1.96 × *SE*.

The genotype-to-phenotype map is calculated by the function GPmap from the result of either linear or multilinear regression. The standard errors of genotypic values are calculated as described above; these errors are also provided in the case of the multilinear regression, though they are probably not meaningful except if the GP map is expected to be actually multilinear: they cover both random departure from the actual GP map and the non-random departure of the GP map from the closest multilinear map. Table 2 compares the GP map that has been used to simulate the dataset, and the GP map inferred from both linear and multilinear regressions. Figure 2 displays the estimates of genotypic values, as well as their confidence intervals, for two different GPmaps (with and without epistasis).

**Figure 3:**
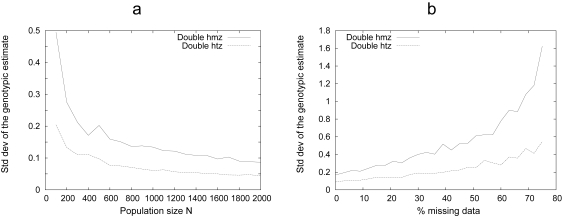
Impact of the quality of the data set on the results. The effect of the population size and the proportion of missing data on the quality of the results is illustrated by the standard deviation of the 2-locus GP map estimates. The amplitude of uncertainties changes with the genotype considered, since the more frequent in the F_2_ population, the better the estimate of the genotypic value. The results for the ‘best’ genotype (i.e. the fully heterozygous (‘htz’) genotype 22) and one of the the ‘worse’ ones (fully homozygous (‘hmz’) 11) are displayed. a: improvement in the precision of the GP map when the size of the population under study is increased. b: effects of substituting (randomly) genotypic information (2 loci, *N* = 500) by missing data. In this example (*Var*(*e*) = 1, additive GP map), fairly good estimates of the genotypic values in a 2-locus GP map requires *N* > 400, and these estimates appear to be quite robust to missing data information. The corresponding script is available in the Appendix.

The precision of the genetic effects and the GP map estimates obviously depends on the quality of the data set. Figure 3a illustrates the effect of increasing the population size on the precision of the genotypic effects, and shows that the improvement is not linear: the gain in precision is weak beyond a population size threshold (in this example, where only 2 loci are involved, *N* ≃ 400). Missing of genotypes (Fig. 3b) affects the results moderately, even at a high frequency (>50%). It is worth noting that sensitivity to missing genotypes depends on the GP map (it is higher when a lot of epistasis is involved). Moreover, the distribution of missing data should not be biased such that the genotypic frequencies are modified significantly.

### Reducing the complexity

**Figure 4:**
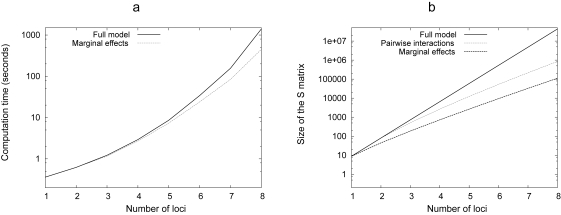
Computational resource requirements. The complexity of the models increases with the number of loci. a) presents the time necessary for the linear regression, with full and marginal-effect models. The test has been performed on a single AMD Athlon 4000 + processor, with the standard R software for Linux (32 bits) and its profiling module (Rprof). Multilinear regression (not shown) is always slower than the corresponding linear regression since this linear regression is first performed to estimate the starting values. b) Increase of the ***S*** matrix size with the number of loci. ***S*** matrix is the largest element in the model, and its size is proportional to the memory necessary to run the program. With a modern desktop PC, it is possible to run regressions up to 10 loci, which is probably beyond the number of genes that can be located in a regular experimental procedure.

By definition, the fully general model provides as many parameters as necessary to describe any GP map (i.e. as many genetic effects as possible genotypes). Increasing the number of loci in the model may therefore lead to an increasing complexity (such as a three or four-way interactions) associated to huge confidence intervals, and possibly overparameterization (more parameters than what can be possibly etimated from the dataset). Moreover, large datasets may lead to computational problems (Fig. 4). The analysis of large and complex GP maps thus requires tools aiming at reducing the number of genetic effects, while keeping as much meaningful information as possible.

Our framework provides two ways of achieving this. The first possibility is to restrict the analysis to a subset of the possible genetic effects, by removing some of them from the analysis. The expected orthogonality of the linear model guarantees that removing any parameter does not impact the other ones, and the hierarchy of genetic effects suggests to get rid of high order effects first, since they are calculated from the residuals of lower-order effects. Dominance, which may not be of interest in all situations, can be removed as well.

**Figure 5:**
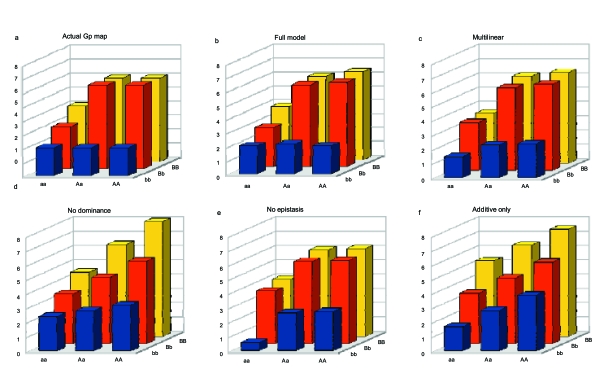
Illustration of the consequences of reducing the complexity of GP maps. An F_2_ population (size *N* = 500, *Var* (*e*) = 1) has been simulated from an arbitrary 2-locus, 2-allele (*a* and *A* at the first locus, *b* and *B* at the other one) GP map (panel a). The inferrence of the GP map from this population with different regression options is displayed in panels b to f (see the Appendix for the corresponding R script). b: Full model (9 parameters), explains 77.7% of the total phenotypic variance; c) multilinear model (6 parameters, 74.3%); d) no dominance (i.e. only additive and additive-by-additive interactions) (4 parameters, 55.9%); e) no epistasis (5 parameters, 70.8%); f ) additive effects only (3 parameters, 54.9%). The full model always performs better (results identical to the actual GP map except sampling effect). The relative performance of the other models obviously depends on the shape of the actual GP map. If the decomposition is orthogonal, a model selection procedure can be performed to make a rational choice among all possible models.

The other possibility is to reduce the number of genetic effects without losing all information about high-order epistasis. This can be achieved through the multilinear regression, which supposes that genetic interactions are proportional to the product of marginal effects. The resulting estimate is the directionality of epistasis, and can be used as a proxy for complex GP maps (Fig. 5).

### Perspectives and conclusion

Model orthogonalization is a general issue in statistics, and is thus not restricted to the decomposition of genetic effects. In any case, getting genetic estimates as independent as possible remains desirable for at least three reasons: (i) it enables straightforward model selection strategies, (ii) it describes the genetic system as average effects of allele substitutions in the sample under study, and (iii) it leads to a proper decomposition of genetic variances. The aim of a research project could however be to focus on a population with properties different from those of the sample under study. Using the change-of-reference tools of NOIA it is possible to transform the obtained estimates from the data into the ones that correspond to the desired reference population, and to obtain the orthogonal decomposition of variance in that population. Furthermore, it is possible to use not only reference populations but also reference individual genotypes. The genetic effects obtained using individual genotypes as reference points make it possible to describe the genetic system as sets of allele substitutions from those individuals. This so-called ‘functional modeling of genetic effects’ enables researchers to use real data to study interesting aspects of evolution such as hybrid incompatibilities and domestication processes (e.g. Le Rouzic et al. 2008).

The NOIA model is perfectly orthogonal at the level of a single locus, whatever the genotypic frequencies. However, it assumes linkage equilibrium (as many previous models for variance decomposition, e.g. Cockerham, 1954; Kempthorne, 1954; Zeng et al. 2005), and is therefore not exactly orthogonal if some genotypes are preferentially associated, even because of random departure from the total equilibrium situation. In practice, a perfectly orthogonal model would lead to results in which the statistical reference point is exactly the mean of the population, and the sum of variance components is exactly the part of variance explained by the model (i.e. the total phenotypic variance minus the residual variance). In any case, simulation results (e.g. Table 1, Fig. 2) show that the small amount of linkage disequilibrium due to unbiased sampling does not affect dramatically the orthogonality of the model.

Further improvement of the NOIA framework include the implementation of a multi-allele model, the current model providing the matrix algebra for only two alleles. This two-allele case is satisfactory in many situations, in which the population under study is the result of a cross between two divergent parental populations. This pattern maximizes the power of gene location, and is thus frequently used, both for domestic populations and natural species surveys. However, many natural populations show a high degree of polymorphism, and would benefit from a more general model. In any case, studying multiple alleles will generate many new genetic parameters to estimate, and would rapidly reach a limit due to the quality and the size of the data set. Practical use of such more general model would thus be doubtful without the development of tools and concepts aiming at simplifying the description of genetic architectures.

Statistical tools such as those described in this contribution aim at providing meaningful and unbiased estimates for many genetic parameters. However, the number of parameters underlying a GP map increases exponentially with the number of genetic factors involved: more loci means more pairwise interactions between loci, and much more high-order interactions. It is likely that most quantitative traits are underlined by large and complex networks of interacting genes, and the development of new technologies in genetics and molecular biology may bring an enormous amount of data on the structure and effects of genetic polymorphisms in such networks. Our ability to extract and summarize information that is relevant for geneticists and evolutionary biologists thus requires the development of tools aiming at manipulating huge datasets not only mathematically, but also conceptually.

## Figures and Tables

**Table 1:** The decomposition of genetic effects in a 2-locus case. The GP map is chosen such as the nine genetic effects are equal to 1 in a perfect F_2_ population. Linear and multilinear regressions are performed on a simulated F_2_ (*N* = 500, *Var*(*e*) = 0.04). The code for genetic effects is indicated as used in the noia package. Genetic effects are indicated by ‘E’, and the reference point follows between brackets (‘pop’ for the statistical effects in the population, ‘P_1_’ for the functional effects in one of the parental populations (genotype ‘1’)). The genetic effects in the P_1_ background are the results of a ‘change-of-reference’ operation (function *geneticEffects*). ‘Var’ is the variance explained by each genetic effect, and the standard errors (Std. err) of genetic effects, as provided by the linear and non-linear regression functions, are indicated. The multilinear regression does not provide any of the classical epistasis components (additive by additive, etc), but a ‘directionality’ parameter. Discrepencies with the expected values of the genetic effects are due to (i) random sampling of phenotypes in the simulated population, and (ii) random linkage disequilibrium in the population that precludes the orthogonality of the model. The decomposition of variance according to the linear model is as follows: additive variance Var(A) = 0.99, dominance variance Var(D) = 0.51, interaction variance Var(I) = 0.53; the sum of genetic variances is thus 2.04, while the expected explained variance from the regression is 1.97. The (small) difference results from covariances due to sampling effects (random linkage disequilibrium). The corresponding script is provided in the Appendix.

Effect	code	E (pop)	Var	Std. err. (pop)	E (P_1_)	E (mult)	Std. err. (mult)
Reference point	..	1.05	1.10	0.0089	0.31	1.03	0.018
Additive, loc 1	a.	1.06	0.57	0.0124	−0.52	1.10	0.025
Dominance, loc 1	d.	1.07	0.29	0.0178	−0.53	1.08	0.036
Additive, loc 2	.a	0.92	0.41	0.0128	−0.51	0.94	0.026
*A* × *A*	aa	0.95	0.22	0.0183	0.99	-	-
*D* × *A*	da	0.97	0.11	0.0257	1.02	-	-
Dominance, loc 2	.d	0.96	0.23	0.0179	−0.58	0.97	0.035
*A* × *D*	ad	1.04	0.14	0.0250	1.04	-	-
*D* × *D*	dd	1.05	0.07	0.0375	1.05	-	-
Directionality (ɛ)	ee	-	-	-	-	0.55	0.025

**Table 2:** GP map estimates. The table displays the GP map estimated from the linear (L) and multilinear (M) regressions described in Table 1. The standard errors are those calculated from the linear model. The precision of the estimates may vary according to the genotype; frequent genotypes (such as the double heterozygote 22) is estimated more accurately than the rare ones (double homozygotes). The GP map from the general model is very close to the actual map, while the multilinear model is constrained and the fit with the expected values may be loose if the real map is not multilinear.

Genotype	Actual map	Estimate (L)	Std. err. (L)	estimate (M)
11	0.25	0.31	0.037	−0.76
21	−0.75	−0.74	0.027	−0.40
31	−0.75	−0.73	0.038	−0.40
12	−0.75	−0.77	0.024	−0.52
22	2.25	2.26	0.018	2.14
32	2.25	2.24	0.025	2.17
13	−0.75	−0.70	0.034	−0.51
23	2.25	2.27	0.025	2.16
33	2.25	2.23	0.035	2.19

## Appendix

library (noia)

### If the package is not installed:

### source ("noia.R")

### To ensure that the results are fully reproducible

set.seed (12346789)

###################### Table 1 and Table 2 #########################

### Build a GP map for 2 loci:

### Order of the genotypes: 11 21 31 12 22 32 13 23 33

map1 <- c (0.25, −0.75, −0.75, −0.75, 2.25, 2.25, −0.75, 2.25, 2.25)

### In this particular GP map, all genetic effects are 1

### in a perfect F2 population

names (map1) <- c ("11", "21", "31", "12", "22", "32", "13", "23", "33") pop1 <- simulatePop (map1, N = 500, sigmaE = 0.2, type = "F2")

### Regressions

linear <- linearRegression (phen = pop1$phen, gen = cbind (pop1$Loc1, pop1$Loc2))

multilinear <- multilinearRegression (phen = pop1$phen, gen = cbind (pop1$Loc1, pop1$Loc2))

### Linear effects, associated variances and stderr

print (linear)

### Multilinear effects

print (multilinear)

### Change of reference: genetic effects in the "11" genotype (parental 1)

print (geneticEffects (linear, ref.genotype = "P1"))

### Variance decomposition

print (varianceDecomposition (linear))

### GP maps

table2 <- cbind (map1, GPmap (linear), GPmap (multilinear) [,1])

colnames (table2) <- c ("Actual", "Lin. effect", "Lin. stderr", "Mult. effects")

print (table2)

####################### Figure 2 #####################

map3 <- c (1, 2, 3, 2, 3, 4, 3, 4, 5)

names (map3) <- c ("11", "21", "31", "12", "22", "32", "13", "23", "33")

map4 <- c (1, 2, 3, 2, 4, 6, 3, 6, 9)

names (map4) <- c ("11", "21", "31", "12", "22", "32", "13", "23", "33")

pop3 <- simulatePop (map3, N = 200, sigmaE = 1, type = "F2")

pop4 <- simulatePop (map4, N = 200, sigmaE = 1, type = "F2")

gpmap3.linear.l1 <- GPmap (linearRegression (phen = pop3$phen, gen = cbind (pop3$Loc1, pop3$Loc2), max. level = 1))

gpmap3.linear.l2 <- GPmap (linearRegression (phen = pop3$phen, gen = cbind (pop3$Loc1, pop3$Loc2), max. level = 2))

gpmap4.linear.l1 <- GPmap (linearRegression (phen = pop4$phen, gen = cbind (pop4$Loc1, pop4$Loc2), max. level = 1))

gpmap4.linear.l2 <- GPmap (linearRegression (phen = pop4$phen, gen = cbind (pop4$Loc1, pop4$Loc2), max. level = 2))

gpmap4.mlinear.l2 <- GPmap (multilinearRegres-sion (phen = pop4$phen, gen = cbind (pop4$Loc1, pop4$Loc2)))

### Figure 2a

print (cbind (map3, gpmap3.linear.l1, gpmap3. linear.l2))

### Figure 2b

print (cbind (map4, gpmap4.linear.l1, gpmap4. linear.l2, gpmap4.mlinear.l2))

####################### Figure 3 #######################

### The GP map does not really matter here

map2 <- c (1, 2, 3, 4, 5, 6, 7, 8, 9)

names (map2) <- c ("11", "21", "31", "12", "22", "32", "13", "23", "33")

### Impact of population size

range <- (100* (1:20))

sd1 <- NULL # Robust genotype estimate (double heterozygote)

sd2 <- NULL # Non-robust genotype estimate (double homozygote)

for (N in range)

{

pop <- simulatePop (map2, N = N, sigmaE = 1, type = "F2")

reg <- linearRegression (phen = pop$phen, gen = cbind (pop$Loc1, pop$Loc2))

gp <- GPmap (reg)

sd1 <- c (sd1, gp["22", 2])

sd2 <- c (sd2, gp["11", 2])

}

cbind (range, sd1, sd2)

### Impact of missing data

range <- 30* (0:25)

sd1 <- NULL # Robust genotype estimate (double heterozygote)

sd2 <- NULL # Non-robust genotype estimate (double homozygote)

for (miss in range)

{

pop <- simulatePop (map2, N = 500, sigmaE = 1, type = "F2")

gen <- cbind (pop$Loc1, pop$Loc2)

gen[sample (1:1000,miss)] <- NA

reg <- linearRegression (phen = pop$phen, gen = gen)

gp <- GPmap (reg)

sd1 <- c (sd1, gp ["22", 2])

sd2 <- c (sd2, gp ["11", 2])

}

cbind (range/1000, sd1, sd2)

####################### Figure 5 ###################################

### Build a custom GP map for 2 loci:

### Order of the genotypes: 11 21 31 12 22 32 13 23 33

map2 <- c (2, 2, 2, 3, 6, 6, 4, 6, 6)

names (map2) <- c ("11", "21", "31", "12", "22", "32", "13", "23", "33")

pop2 <- simulatePop (map2, N = 500, sigmaE = 1, type = "F2")

### Full model

full <- linearRegression (phen = pop2$phen, gen = cbind (pop2$Loc1, pop2$Loc2))

### Multilinear regression

mult <- multilinearRegression (phen = pop2$phen, gen = cbind (pop2$Loc1, pop2$Loc2))

### Without dominance

wo.dom <- linearRegression (phen = pop2$phen, gen = cbind (pop2$Loc1, pop2$Loc2), max. dom = 0)

### Without epistasis

wo.epi <- linearRegression (phen = pop2$phen, gen = cbind (pop2$Loc1, pop2$Loc2), max. level = 1)

### Only additive effects

add <- linearRegression (phen = pop2$phen, gen = cbind (pop2$Loc1, pop2$Loc2), max.level = 1, max.dom = 0)

### Comparing the maps

comp <- cbind (map2, GPmap (full) [,1], GPmap (mult) [,1], GPmap (wo.dom) [,1], GPmap (wo.epi) [,1], GPmap (add) [,1]) colnames (comp) <- c ("true", "full", "mult", "wo. dom", "wo.epi", "add")

print (comp)
